# Variation in Methyl Jasmonate-Induced Defense Among Norway Spruce Clones and Trade-Offs in Resistance Against a Fungal and an Insect Pest

**DOI:** 10.3389/fpls.2021.678959

**Published:** 2021-05-24

**Authors:** Adriana Puentes, Tao Zhao, Lina Lundborg, Niklas Björklund, Anna-Karin Borg-Karlson

**Affiliations:** ^1^Department of Ecology, Swedish University of Agricultural Sciences, Uppsala, Sweden; ^2^Man-Technology-Environment Research Centre, Örebro University, Örebro, Sweden; ^3^Department of Chemistry, Organic Chemistry, KTH, Royal Institute of Technology, School of Chemical Science and Engineering, Stockholm, Sweden; ^4^Department of Chemical Engineering, Mid Sweden University, Sundsvall, Sweden

**Keywords:** *Ceratocystis polonica*, conifer resistance, *Endoconidiophora polonica*, forest pest, genetic correlations, *Hylobius abietis*, resistance trade-offs, terpene chemistry

## Abstract

An essential component of plant defense is the change that occurs from a constitutive to an induced state following damage or infection. Exogenous application of the plant hormone methyl jasmonate (MeJA) has shown great potential to be used as a defense inducer prior to pest exposure, and could be used as a plant protection measure. Here, we examined (1) the importance of MeJA-mediated induction for Norway spruce (*Picea abies*) resistance against damage by the pine weevil *Hylobius abietis*, which poses a threat to seedling survival, and infection by the spruce bark beetle-associated blue-stain fungus *Endoconidiophora polonica*, (2) genotypic variation in MeJA-induced defense (terpene chemistry), and (3) correlations among resistance to each pest. In a semi-field experiment, we exposed rooted-cuttings from nine different Norway spruce clones to insect damage and fungal infection separately. Plants were treated with 0, 25, or 50 mM MeJA, and planted in blocks where only pine weevils were released, or in a separate block in which plants were fungus-inoculated or not (control group). As measures of resistance, stem area debarked and fungal lesion lengths were assessed, and as a measure of defensive capacity, terpene chemistry was examined. We found that MeJA treatment increased resistance to *H. abietis* and *E. polonica*, but effects varied with clone. Norway spruce clones that exhibited high constitutive resistance did not show large changes in area debarked or lesion length when MeJA-treated, and vice versa. Moreover, insect damage negatively correlated with fungal infection. Clones receiving little pine weevil damage experienced larger lesion lengths, and vice versa, both in the constitutive and induced states. Changes in absolute terpene concentrations occurred with MeJA treatment (but not on proportional terpene concentrations), however, variation in chemistry was mostly explained by differences between clones. We conclude that MeJA can enhance protection against *H. abietis* and *E. polonica*, but the extent of protection will depend on the importance of constitutive and induced resistance for the Norway spruce clone in question. Trade-offs among resistances do not necessarily hinder the use of MeJA, as clones that are constitutively more resistant to either pest, should show greater MeJA-induced resistance against the other.

## Introduction

Plant resistance to biotic threats is mediated through defensive traits that are present at all times (constitutive defense), but also through those triggered once damage or infection is perceived (induced defense). Constitutive defenses provide a first line of protection that can deter or stop attackers, while induced defenses strengthen these effects making plants unsuitable hosts and decrease the likelihood of further attack (Karban and Myers, 1989; Agrawal, 1999). In conifers, quantitative and qualitative changes to constitutive conditions are rapidly initiated upon mechanical wounding, insect feeding and fungal infection. Interestingly, defenses can even be mobilized earlier and prior to damage, as for example recently shown for the effects of insect egg deposition on *Pinus sylvestris* (Bittner et al., 2019). These responses include secondary resin production and traumatic resin duct formation, synthesis of new phenolics, lignification of fibers, and initiation of wound periderm (Franceschi et al., 2005). The difference between the constitutive and induced state is referred to as “inducibility,” and represents a key functional trait of defensive investment and effective resistance to specific enemies (Cipollini and Heil, 2010). Indeed, the essential contribution of induced responses to effective conifer resistance has been demonstrated for bark-beetle-fungi complexes (e.g., Krokene, 2015; Raffa et al., 2017), infection by fungal pathogens (e.g., Danielsson et al., 2011; Ganthaler et al., 2017) as well as weevil pests (e.g., Zas et al., 2014; Whitehill et al., 2019). Host colonization by many of these attackers impairs water and nutrient transport, and result in tree death if induced responses fail to hamper damage levels (Kolosova and Bohlmann, 2012). Thus, understanding the extent of inducibility is essential for improving tree survival, for example within forest protection, and to reveal ecologically-relevant tree properties that mediate interactions.

The extent of effective resistance against coniferous pests, achieved through constitutive and/or induced resistance, has been largely examined separately for most pest organisms without consideration on how they affect each other. Resistant individuals are those that can stop or deter attackers, and thus effectively reduce their damage or infection levels relative to susceptible individuals. In particular, little is known about the relationship between resistance to insects and fungal pathogens that are not mutually associated (i.e., those that do not co-occur and depend on each other for host colonization), and if the relative importance of defense components varies or shifts with respect to each pest species (Eyles et al., 2010; Raffa et al., 2020). Given that effective resistance against fungal and insect pests may or may not involve different defense pathways (e.g., jasmonic acid or salicylic acid signaling pathways), the consequences of responses induced by these pests can be antagonistic or complementary to each other (Rostás et al., 2003; Beckers and Spoel, 2006; Eyles et al., 2007). For example, responses to feeding by the jack pine budworm (*Choristoneura pinus pinus*) have been shown to also mediate effective resistance against a fungal pathogen (*Grosmannia clavigera*) in *Pinus banksiana* (Colgan and Erbilgin, 2010). On the other hand, attack by the silver fir wooly adelgid (*Dreyfusia nordmannianae*) makes *Abies nordmanniana* more susceptible to infection by the fungus *Neonectria neomacrospora* (Xu et al., 2018). Thus, identifying potential correlations among defense components to different pests is essential to understanding the outcome of interactions.

The presence or absence of trade-offs among defense components against specific pests, and the consequences for effective resistance, can vary depending on plant genotype or species in question (e.g., Koricheva et al., 2004; Erb et al., 2011; Moreira et al., 2014; Hahn and Maron, 2016; Shikano et al., 2017). For instance, not all genotypes respond equally to attack by the same pest in conifers, as they can vary, e.g., in their allocation to constitutive and induced defense (e.g., Ott et al., 2011; Sampedro et al., 2011; Villari et al., 2014; Moreira et al., 2016; Pimentel et al., 2017; Reglinski et al., 2019). Moreover, the relative importance of constitutive and induced defense is also dependent on whether the species examined is fast- or slow-growing, or its degree of competitive ability (Endara and Coley, 2011; Kempel et al., 2011; Moreira et al., 2014). Differences in allocation to defense strategies can result in trade-offs among constitutive and induced resistance (e.g., Rasmann et al., 2011). Depending on which strategy is most important for deterring the attacker in question, this could in turn result in negative correlations among resistances to each pest. Understanding such variation in resistance trade-offs is especially important for conifer breeding programs and novel plant protection tools aimed at enhancing intrinsic tree defenses. Without knowledge of such correlations, breeding may select for genotypes that exhibit opposing susceptibility to, e.g., fungal and insect pests. Thus, uncovering genetic relationships among defenses is crucial to producing improved tree material that can effectively resist various pests (Telford et al., 2015).

From a plant protection perspective, exogenous application of phytohormones such as jasmonic acid and its methyl ester (methyl jasmonate) has been proposed as a tool to prime or induce defenses associated with conifer resistance to insects and fungal pests (Holopainen et al., 2009; Zas et al., 2014; Mageroy et al., 2020a). Treatment with methyl jasmonate (MeJA), for example, induces the formation of traumatic resin ducts and synthesis of terpene and phenolic-based compounds (Krokene et al., 2008; López-Villamor et al., 2020). These MeJA-mediated changes have been shown to increase resistance against bark-feeding insect pests like the pine weevil *Hylobius abietis* (Heijari et al., 2005; Zas et al., 2014; Fedderwitz et al., 2016; Chen Y. et al., 2020), and the spruce bark beetle-blue stain fungus complex *Ips typographus*-*Endoconidiophora polonica* (Zhao et al., 2011; Schiebe et al., 2012). However, responses to MeJA can vary with plant genotype (Zeneli et al., 2006; Moreira et al., 2013; López-Goldar et al., 2018), with some genotypes showing greater or lesser levels of inducibility relative to their constitutive state. Therefore, to tackle challenges posed by multiple pests it is not only of interest to identify trade-offs in defense, but also those genotypes that respond strongly to elicitors such as MeJA and effectively increase their resistance. This knowledge allows us to better predict and understand the outcome of conifer interactions with various pests, and implement long-lasting and sustainable plant protection strategies.

In this study, we conducted an experiment to examine genotypic variation in defense inducibility, in terms of terpene chemistry, and the importance of induction for insect and fungal pathogen resistance in Norway spruce (*Picea abies*). We examined variation between clones in MeJA-induced responses, and to damage by the pine weevil (*H. abietis*) and a virulent blue-stain fungus (*E. polonica*) associated with the spruce bark beetle. The pine weevil represents the main threat to newly planted conifer plants in Europe (Långström and Day, 2004) as it can feed on the phloem and bark of seedlings, causing stem girdling and high mortality rates. The necrotrophic blue-stain fungus *E. polonica* plays a key role in mediating tree death following spruce bark beetle attacks, which are a major threat to mature Norway spruce in Europe (Krokene, 2015; Hlásny et al., 2019). Thus, the pine weevil and blue-stain fungus pose large threats to tree survival but at different developmental stages. Resistance to these economically-important threats has been examined separately, and the relationship among resistance to insect damage and fungal infection is not known. Being resistant to the pine weevil early in life, for example, could come at the cost of being susceptible to fungal infection later in life and vice versa. Such trade-offs are important from an ecological perspective, but also from a plant breeding perspective as there are ongoing efforts to produce genetically improved tree material that is resistant to various pests. Here, we aimed to investigate the correlation among constitutive and induced resistance to an herbivorous insect and a pathogenic fungus. More specifically, we aimed to answer:

1. Does MeJA treatment influence the terpene chemistry of Norway spruce and its resistance against the pine weevil *H. abietis* and the blue-stain fungus *E. polonica*?
2. Does the effect of MeJA treatment on terpene chemistry and resistance against *H. abietis* and *E. polonica* vary among different clones of Norway spruce?
3. Does resistance against the pine weevil *H. abietis* and blue-stain fungus *E. polonica* exhibit trade-offs?

To answer these questions, we used a clonal set-up where we compared chemical defenses (terpenes) and resistance of MeJA-induced and non-induced plants of the same Norway spruce clones. Resistance to the insect and fungus pests were investigated separately (in different individuals of the same clones) in a semi-field experiment. We quantified stem area debarked by the pine weevil and fungal growth (lesion length) as measures of plant resistance, with resistant plants being those receiving little to no insect damage or shorter lesion lengths. We quantified terpene chemistry as a measure of defense, and refer to it as defense or defensive chemistry throughout. The use of clones facilitates correlations among resistances to be estimated, and to tease apart the relative importance of constitutive and induced resistance against each attacker.

## Materials and Methods

### Plant Material and Methyl Jasmonate Treatments

In August 2011, cuttings were made from nine clones (clone identification numbers: 1 to 9 hereafter) of Norway spruce [*P. abies* (L.) Karst.]. These clones originated from the clonal archive material produced by The Forestry Research Institute of Sweden (Skogforsk, series S21K0420-), as part of their breeding trials for Norway spruce. Cuttings were individually planted in 6.5 cm-sized pots, and allowed to grow at the growing facilities in Skogforsk (Ekebo, 55.9°N; outside of Svalöv in southern Sweden) first, and later at the Swedish University of Agricultural Sciences (SLU, Uppsala, 59°49′N, Sweden) until the start of the experiment in the summer of 2014. By then, plants had reached an average height of 32.6 cm (standard error: ±0.4), and a diameter of 6.3 mm (±0.08; Supplementary Figure 2).

To examine constitutive and MeJA-induced responses in Norway spruce clones, plants from each clone were assigned to either a control or a MeJA treatment group. According to the Krutzsch index (Krutzsch, 1973), clones were at a similar developmental stage (between 0 and 3, Supplementary Table 1) when MeJA was applied. We chose two concentrations of MeJA (25 and 50 mM) based on previous studies in our group and on the size of the plants. Smaller Norway spruce plants (average height: 20 cm, average diameter: 3 mm) have often been treated with concentrations ranging from 5 to 15 mM MeJA (Fedderwitz et al., 2019; Chen Y. et al., 2020). Larger and thicker plants (average heights above 25 cm, and diameters above 5 mm) as those used in our experiment, have been treated with higher concentrations (25 mM MeJA, Lundborg et al., 2016b; 50 mM MeJA, Fedderwitz et al., 2016). For the pine weevil experiment, 20 replicates per clone (*n* = 9 clones) were included in each of the three treatments (0, 25 and 50 mM MeJA). However, clone number 5 had three individuals less than all the other clones from the beginning, and plants in the 50 mM MeJA treatment for clone number 1 were not planted in the field (see Semi-field experiment description). The total sample sizes for 0, 25, and 50 mM MeJA treatments were 180, 177, and 160 plants, respectively. For the fungus-inoculation experiment, 5 plants per clone were included in each of the three treatments. Clone numbers 3 and 6 had one less plant, and clones 4 and 7 had two less plants from the beginning, and for clone number 1 plants in the 50 mM MeJA were not planted in the field. The total sample sizes for 0, 25, and 50 mM MeJA treatments were 43, 45, and 36 plants, respectively.

Treatment of Norway spruce plants with MeJA followed Zas et al. (2014). Briefly, MeJA (95%, Sigma-Aldrich, ref. 392707) was first dissolved in ethanol, then deionized water was added to this mixture to achieve a final ethanol concentration of 2.5% (v:v). The solution was shaken vigorously until a uniform milky emulsion was obtained, and then transferred to a spraying bottle (0.5 L plastic bottle; Part No. 62526011, Canyon, United Kingdom). Spraying was conducted so that the solution reached and covered the entire plant in each pot. Each plant received approximately 1 ml of either 25 or 50 mM MeJA solution. The control group (0 mM MeJA) was treated in the same way but only with carrier solution (deionized water and ethanol). Plants were treated twice, once on April 28th and then again on May 14th, 2014. To avoid any potential defense induction of neighboring non-treated controls, MeJA-treated and control plants were kept separately from the first application of MeJA until planting occurred.

### Semi-Field Experiment

To examine constitutive and MeJA-induced resistance against the pine weevil and the blue-stain fungus, a semi-field experiment was set up. On May 21st, 2014 plants were planted on a 1-year-old clear-cut situated in a forest dominated by Scots pine (*P. sylvestris*), located near Uppsala, Sweden. The plants were randomly assigned to positions in rows with a spacing of 0.1 m between the plants in the same row, and 0.1 m between the rows. Planting was done all in 1 day and plants were planted with their entire soil plug in the clear-cut (see Supplementary Figure 1 for the soil plug). Planting was done using a standard cylindrical planting tube (Pottiputki, Finland, 75 mm). To minimize planting stress, we kept soil plugs in a bucket with water before planting them. Once the planting hole was made with the planting tube, we poured water into the hole to moisten the soil and minimize the risk of drought stress. Plants in all treatments and enclosures were treated the same way.

Plants intended for exposure to pine weevils were planted separately from plants intended for fungal inoculations. In the same clear-cut, four enclosures (see description below) were built for the pine weevil experiment, enclosing 127, 130, 130, and 130 plants each, and one enclosure with two sub-plots for the 250 plants included in the fungal inoculation experiment. All MeJA treatment × clone combinations were represented in each enclosure, but replicate numbers for each combination varied across enclosures. However, the plants from the 50 mM treatment of clone 1 were never included in the experiment due to MeJA-treatment-related damage, i.e., needles turning brown before the start of the field experiment. Plant height, basal stem diameter and top shoot length were measured for each plant before the start of the experiments.

#### Pine Weevil Experiment

To investigate resistance to pine weevil damage, weevils were released in the enclosures containing the group of plants intended for this purpose. Approximately 300 pine weevils were released on July 1st, 2014 in each of the four enclosures (48 days after the second MeJA treatment occurred). The enclosures were about 0.2 m high, and prevented pine weevils from escaping and reaching the experimental plants intended for fungal inoculations. Each enclosure consisted of a wooden framework placed around each group of plants (but open across the top). The inner edges of the wooden enclosure were covered with a plastic film, and this plastic film was painted with polytetrafluoroethylene (Fluon^®^, Blades Biological Ltd., Cowden Edenbridge, Kent, United Kingdom). This created a slippery surface preventing the weevils from climbing over the enclosure. The pine weevils that were released into these enclosures had been collected at the same clear-cut earlier in the spring, and kept in rearing boxes with access to food (freshly-cut conifer branches) and water until the start of the experiment. After release, damage inflicted by pine weevils on each plant was measured as the stem area debarked (cm^2^), and was recorded during July 23rd–24th, 2014. Area debarked was measured as the sum of the areas of each wound inflicted by the pine weevil. A template with different area sizes illustrated on millimeter paper, was used for calculating the area of each wound. Only the area, and not the depth of the wound, was measured. Enclosures were included as blocks in the statistical analyses.

#### Blue-Stain Fungus Experiment

The strain of the blue stain fungus *E. polonica*, which was used for inoculations, was NFLI 1993–208/115. It was obtained from the culture collection of the Norwegian Institute for Bioeconomy Research in Ås, Norway. The strain was isolated from a Norway spruce log inoculated with the bark beetle *Polygraphus poligraphus* L. (Krokene and Solheim, 1996). This *E. polonica* isolate has been used in many of our previous studies (e.g., Zhao et al., 2010, 2011; Axelsson et al., 2020), it grows well on Norway spruce and has shown consistent virulence across our studies. The fungal strain was maintained on malt agar (2% malt, 1.5% agar) at 4°C, and transferred to fresh malt agar and cultivated at 25°C in darkness for 7-10 days, before the start of the experiment.

To investigate resistance against the blue-stain fungus, plants in the different MeJA treatments (0, 25 or 50 mM) were further assigned to two treatments: fungal or no fungal inoculation (control) group. On July 1st and 2nd, 2014 (48-49 days after the second MeJA treatment occurred) the lower part of the stem of plants in the fungal inoculation group was inoculated with *E. polonica*. Using a 4 mm cork borer, a phloem plug (about 1.5-2.0 mm in depth) was removed from each plant and an agar plug with *E. polonica* inoculum was in turn introduced. For plants in the control group, an agar plug not containing *E. polonica* was introduced instead. The *E. polonica* inoculum or the non-infected agar plug was fixed to the stem with Parafilm®. On August 29th, 2014 plants were harvested for analyses, i.e., about 8 weeks after the inoculation. The blue-stain fungus experiment was ended at a later date than the pine weevil experiment, given that the insect feeds at a faster pace than fungal lesions occur. Lesion lengths of *E. polonica* in the phloem were assessed by removing the outer bark upward from the inoculation point. A surgical knife was used for the removal of the bark and the length of the lesions was measured using a ruler. Only for plants in the 0 and 50 mM MeJA treatments, we cut the plants into parts and kept only ten centimeters of the entire stem (which included the inoculated area), and froze these stem pieces in an −80°C freezer until chemical analyses were conducted.

### Chemical Analyses

The terpene chemistry of plants in the blue-stain fungus experiment (*E. polonica-*inoculated and control group) which had received 0 and 50 mM MeJA treatment, was quantified to investigate the effect of MeJA on plants’ terpene production. Note that for clone 1, plants in the 50 mM were never included in the experiment, so for this clone plants in the 25 mM MeJA treatment were used instead. No plants in the insect enclosure experiment were harvested for chemical analyses.

Stem pieces were taken from the freezer after 5 months, and two phloem samples were removed from stems using a surgical knife. To examine the chemical effects of the MeJA treatment *per se* (non-fungus inoculated MeJA treated vs. untreated plants), phloem was collected from a “control zone” located 5 cm below and on the opposite side of the stem from the “reaction zone” (inoculation area). Further, to also be able to analyze the induced defense caused by fungus inoculation, phloem was collected from directly below the inoculation area. This method of comparing tissue from the “inoculation area” and a “control area” from the opposite side of the stem, is usually used in older trees with thicker trunks (e.g., Axelsson et al., 2020). Since we used thinner trees in our experiment, we cannot be completely sure that fungal infection does not affect the opposite side of the stem bark. Samples of approx. 1.5 cm^2^ in size were chopped into smaller pieces (following standard protocol as described in Persson et al., 1993; Axelsson et al., 2020), and each was immediately placed (using tweezers) in a 2-ml glass vial with solvent. Phloem samples were extracted in 0.5 mL n-hexane (VWR, Ref no. 601-037-00-0) containing 0.05 mg mL^–1^ of internal standard (pentadecane; Lancaster synthesis, Alfa Aesar). Extraction time was 48 h, and after this time, extracts were transferred to new 2-ml glass vials. These were stored at −30°C until analysis using a gas chromatograph-mass spectrometer (GC-MS) was conducted. The dry weight of the extracted phloem pieces was measured after 5 h at 80°C.

The separation and identification of volatiles was made on a 2DGC-MS Agilent instrument (7890A GC; 5975C MS), equipped with a DB-5 column followed by a Cyclodextrin-β column (both Agilent; 30 m, ID 0.25 mm, and film thickness 0.25 μm). Samples were injected splitless into an injector temperature of 250°C, isothermal, and with a purge time of 1 min. The GC oven program started at 40°C, and was kept isothermal for 3 min, followed by a temperature ramp of 3°C min^–1^, up to 100°C, followed by a second ramp of 5°C min^–1^ up to 250°C, and then isothermal for 1 min. The transfer line temperature to the second GC was isothermal at 40°C.

The temperature program in the second GC was isothermal at 58°C for 50 min, then ramped from 100°C min^–1^ up to 200°C, and kept for 3.5 min. On the second column, the enantiomers of α-pinene, β-pinene and limonene were separated. The first two substances were cut from the first to the second column between 8 and 14 min, and evaluated on m/z 93 (their most abundant fragment). On the first column, the coeluting limonene and β-phellandrene were quantified on the amount of m/z 68 compared to limonene standard (β-phellandrene does not give this fragment). These were in the same run as the other chiral compounds, cut from 15 to 18 min on GC1, for limonene to be evaluated on m/z 68 on GC2.

The terpene hydrocarbons were identified by comparing retention times and mass spectra with available authentic standards, or by comparing retention indexes (RIs) and mass spectra with Massfinder 3 (Hochmuth Scientific Consulting, Germany) and the reference libraries of NIST (National Institute of Standards and Technology, United States). The absolute amounts of terpenes were calculated relative to the internal standards, and expressed as μg g^–1^ dry wt. The relative amounts of terpenes were calculated as the ratio of the area of each peak to the sum of all the areas of terpene hydrocarbons in a defined GC fraction, and expressed as percentages.

### Statistical Analyses

All analyses were conducted in R version 4.0.0 (R Core Team, 2020) using R studio version 1.1.463 (RStudio Team, 2020), and figures were plotted using *ggplot2* (Wickham, 2016). To examine constitutive and MeJA-induced defense and resistance against *H. abietis* and *E. polonica*, and if this varies among clones, we fitted various linear models using either the *lm* (The R stats package, R Core Team, 2020) or the *glmmTMB* functions in R (*glmmTMB* package, Brooks et al., 2017). For pine weevil damage (response variable: stem area debarked), we fitted a *glmmTMB* model with a negative binomial distribution that included MeJA treatment (3 levels: 0, 25, and 50 mM), clone (*n* = 8, since clone 1 had no individuals in the 50 mM MeJA treatment), and the interaction of MeJA and clone, with enclosure (*n* = 4) and plant height (continuous covariate) as fixed factors. Model fit was explored with the *DHARMa* package (Hartig, 2020) and no data transformations were necessary. Significance of main effects and interactions was tested using analysis of deviance with the *Anova* function (*car* package, Fox and Weisberg, 2019). For fungal infection (response variable: lesion length), an *lm* model that included MeJA treatment (3 levels: 0, 25, and 50 mM), clone (*n* = 8), the interaction of MeJA and clone, and sub-plot (*n* = 2) within one enclosure as fixed factors. Lesion length was log-transformed to meet model assumptions. Significance of main effects and interactions was tested using an *F*-test with the *Anova* function. Contrasts among treatment means were conducted using the *emmeans* function (*emmeans* package, Lenth et al., 2020). Note, however, that we present visually results for models that include only two MeJA levels (0 and 50 mM, and 0 and 25 mM for clone 1, *n* = 9 clones) for both area debarked and lesion length (see motivation in Results section).

To examine any potential trade-offs between resistance to insect and fungal damage, we conducted Pearson’s product-moment correlations using the *cor.test* function in R (The R stats package, R Core Team, 2020). To examine the correlation between constitutive resistances, we correlated estimated means for each clone (from the models described above) for area debarked and lesion length in the control group (0 mM MeJA). To examine the correlation in MeJA-induced resistance, we correlated the coefficients of the change between 0 and 50 mM (i.e., slope of the reaction norms) for each clone, from the models described above for area debarked and lesion length. Using these estimates, we also conducted Pearson’s product-moment correlations between terpene compounds and insect damage or fungal infection. Mean estimates of area debarked or lesion length were correlated to the average absolute terpene amounts of each compound (or the sum of all terpenes) per clone, both under the constitutive and induced state and (0 and 50 mM MeJA respectively) but without fungal inoculation. These bivariate correlations were conducted separately for each terpene compound and area debarked or lesion length.

For analysis of the chemical data, terpenes were selected with a 2% limit in relative amounts, in at least 3 replicates and then normalized to 100%. In our experiment, Norway spruce clones previously treated with 0 or 50 mM MeJA were subsequently inoculated with an agar plug (no fungus) or *E. polonica*. Thus, both MeJA treatments are represented in the agar and fungus-inoculated plant groups. Since we were interested in examining the effect of MeJA separately from that of fungal infection on terpene chemistry, we conducted individual analyses for those plants receiving an agar plug or *E. polonica*. The analyses described below were conducted for each of these two plant groups. The effects of MeJA treatment (0 or 50 mM), Norway spruce clone (*n* = 9), and their interaction, were examined with a multivariate analysis of variance (MANOVA; *adonis* function, *vegan* package version 2.5-6, Oksanen et al., 2019) on the absolute amounts of terpene compounds. We also fitted the same model but using instead relative amounts as a response variable (amount of each terpene compound was divided by the sum of total terpenes for that sample). In other words, this model examined proportional changes in terpene chemistry. To visualize the effect of treatment and clone on the chemical profile, dimensions of the dataset acquired from the GC-MS analysis were reduced by Principal Component Analysis (PCA), also using the *vegan* package in R. Loadings were obtained to examine the contribution of each compound to the PCAs first and second axes of variation.

## Results

We found that MeJA treatment decreased the extent of pine weevil damage and blue-stain fungus infection on Norway spruce clones, which were separately exposed to these two pests. Plants treated with 25 or 50 mM MeJA showed significantly lower levels of insect damage and shorter lesion lengths relative to control plants (Supplementary Table 2). However, clones responded similarly to 25 and 50 mM MeJA, with no significant differences among these treatments for insect or fungal damage (Supplementary Table 2). Given this lack of difference and that samples for terpene chemistry were only taken from Norway spruce clones receiving 0 and 50 mM MeJA, we present results based on models that included only these two levels of MeJA. Note that for Norway spruce clone number 1, plants in the 50 mM MeJA group were never planted in the field experiment due to needle browning (see section “Materials and Methods”), so results for the 25 mM MeJA treatment are presented instead.

**FIGURE 1:**
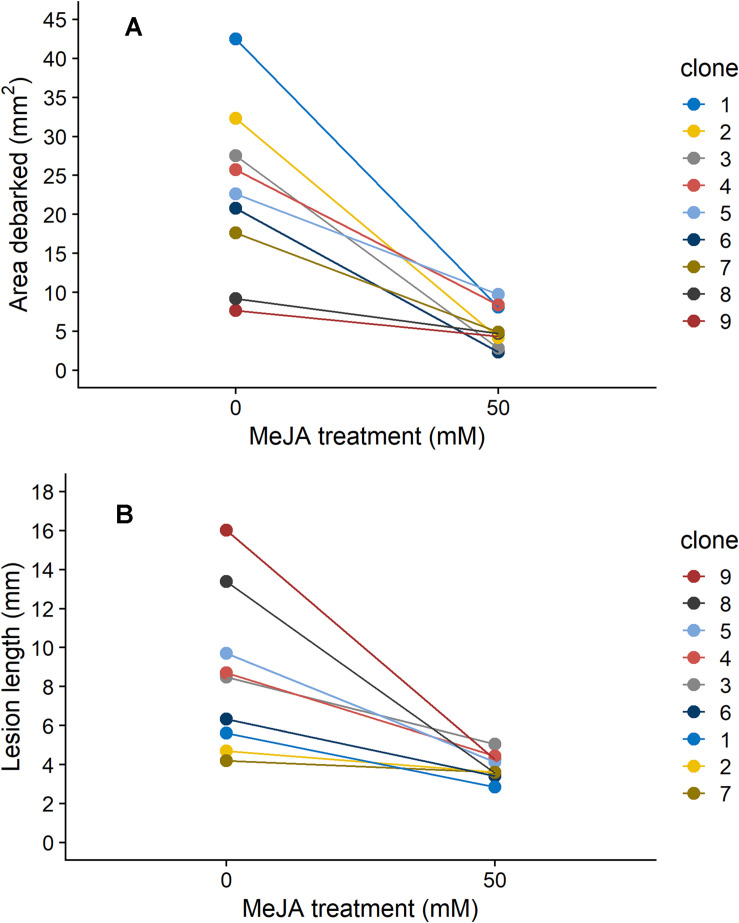
Reaction norms depicting the change in **(A)** area debarked (mm^2^) by pine weevils (*H. abietis*) and **(B)** lesion lengths (mm) inflicted by the blue-stain fungus (*E. polonica*) on Norway spruce (*P. abies*) clones (1–9) treated with 0 or 50 mM methyl jasmonate (MeJA). Estimated means (from models presented in Table 1) for each clone are shown. For clone 1, plants in the 50 mM were not included in the experiment (see section “Materials and Methods”), so results for the 25 mM MeJA treatment are presented instead. The order in which each clone’s reaction norm appears in the graph is reflected in the clone number legend for each panel. Estimates ± standard error for area debarked and lesion length, per clone and treatment combination, can be found in Supplementary Table 3.

Norway spruce plants that were treated with 50 mM MeJA received on average 76% less pine weevil damage, and experienced 55% shorter lesion lengths compared to those in the control group (Figure 1). However, the decrease in insect damage and fungal infection observed for plants in the MeJA treatment tended to differ among clones (Figure 1). For area debarked, there was a statistically significant MeJA × clone interaction (Table 1), while for lesion length it was close to significant at the *P* < 0.1 level (Table 1). Overall, there was greater variation among clones in the amount of pine weevil damage and fungal infection when plants were not induced (0 mM MeJA) relative to when they were MeJA-induced, especially for lesion length (Figures 1A,B, compare 50 mM MeJA for the two variables). In other words, clones responded more similarly to MeJA treatment in terms of lesion length than area debarked. Nonetheless, some clones such as numbers 2 and 7 showed little change in lesion length when treated with MeJA (Figure 1B).

**TABLE 1:** Analysis of deviance (Chisq, Chi-square Wald statistic; DF, degrees of freedom; and *P*, *p*-value) and Analysis of variance (SS, Sum of Squares; DF, degrees of freedom; *F*, *F*-value; and *P*, *p*-value) results from models examining the effects of MeJA (MJ: 0, 50 mM) on area debarked (mm^2^) by pine weevils (*H. abietis*) or lesion lengths (mm) inflicted by the blue-stain fungus (*E. polonica*) on Norway spruce (*P. abies*) clones.

Variable	Source	Chisq	DF	*P*	Variable	Source	SS	DF	*F*	*P*
Area debarked (mm^2^)	MJ	101.3	1	**<0.0001**	Lesion length (mm)	MJ	10.8	1	50.1	**<0.0001**
	Clone	39.2	8	**<0.0001**		Clone	5.8	8	3.4	**0.003**
	MJ × clone	18.5	8	**0.02**		MJ × clone	3.1	8	1.8	0.08
	Plant height	11.9	1	0.0006		Block	0.1	1	0.5	0.5
	Block	52.6	3	<0.0001		Residuals	13.9	65		

*Models included the main effect and interaction of MeJA (MJ) and clone, experimental enclosure or sub-plot for lesion length (Block), and plant height as a continuous covariate (for area debarked only). Significant effects are in bold (*P* < 0.05).*

**FIGURE 2:**
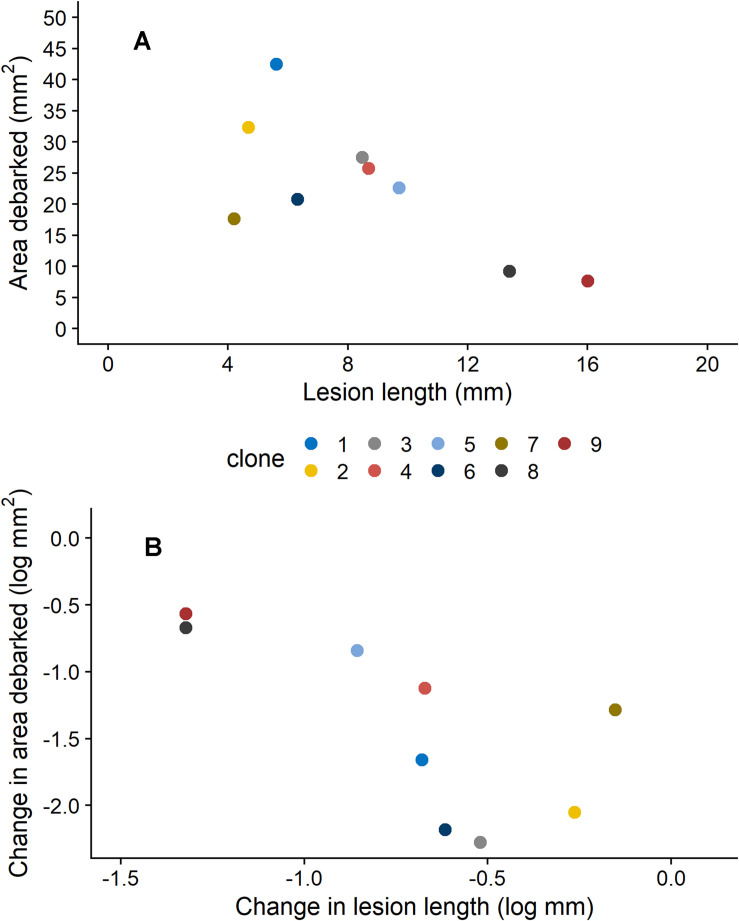
Relationship between **(A)** estimated means (from models presented in Table 1) for area debarked (mm^2^) by pine weevils (*H. abietis*) and lesion lengths (mm) inflicted by the blue-stain fungus (*E. polonica*) on Norway spruce (*P. abies*) clones (1–9) treated with 0 mM methyl jasmonate (MeJA), and **(B)** the change in area debarked (log mm^2^) and lesion length (log mm; i.e., slope coefficients of reaction norms) that occurred for each clone between the 0 and 50 mM MeJA treatments. Clones in the 0 mM MeJA that received little pine weevil damage experienced longer fungal lesion lengths, and vice versa [panel **(A)**]. Clones that exhibited a minor difference (i.e., decrease) in area debarked between the 0 and 50 mM MeJA treatment, experienced a greater change (i.e., decrease) in fungal lesion lengths between these two treatments [panel **(B)**]. For clone 1, plants in the 50 mM were not included the experiment (see section “Materials and Methods”), so results for the 25 mM MeJA treatment are presented instead. Estimates ± standard error for area debarked and lesion length, per clone and treatment combination, can be found in Supplementary Table 3.

We also found that insect damage and fungal infection exhibited a negative relationship to each other, when estimates for each clone were compared. For plants that were not induced (0 mM MeJA), mean estimates of area debarked and lesion length for each clone negatively correlated with each other (Figure 2A; Pearson’s product-moment correlation coefficient: −0.71, 95% Confidence intervals: −0.93, −0.08, *t* = −2.7, DF = 7, and *P* = 0.033). Likewise, we found a negative correlation between the effect of MeJA treatment on lesion length and area debarked (Figure 2B; Pearson’s product-moment correlation coefficient: −0.70, 95% Confidence intervals: −0.93, −0.06, *t* = −2.6, DF = 7, and *P* = 0.037). In other words, we found a negative relationship between the estimates of the change in area debarked and lesion length that occured from the 0 to the 50 mM MeJA treatment (reaction norms observed in Figure 1). Norway spruce clones showing the largest reduction in area debarked following MeJA treatment, showed a smaller reduction in lesion length and viceversa. We also found a few negative bivariate correlations between mean area debarked per clone and each teperne compound, but only when plants had been treated with 50 mM MeJA (Supplementary Table 7). Moreover, mean estimates of lesion lengths per clone positively correlated with some terpene compounds, but only in the constitutive state (0 mM MeJA) (Supplementary Table 7). In line with other studies, we found that MeJA negatively affected plant growth in terms of apical shoot length, total plant height and stem diameter (Supplementary Figure 2 and Supplementary Table 4). On average, the the apical (top) shoot length decreased with 22%, the total plant height with 3%, and the stem diameter with 2% when plants were MeJA-treated relative to controls.

**FIGURE 3:**
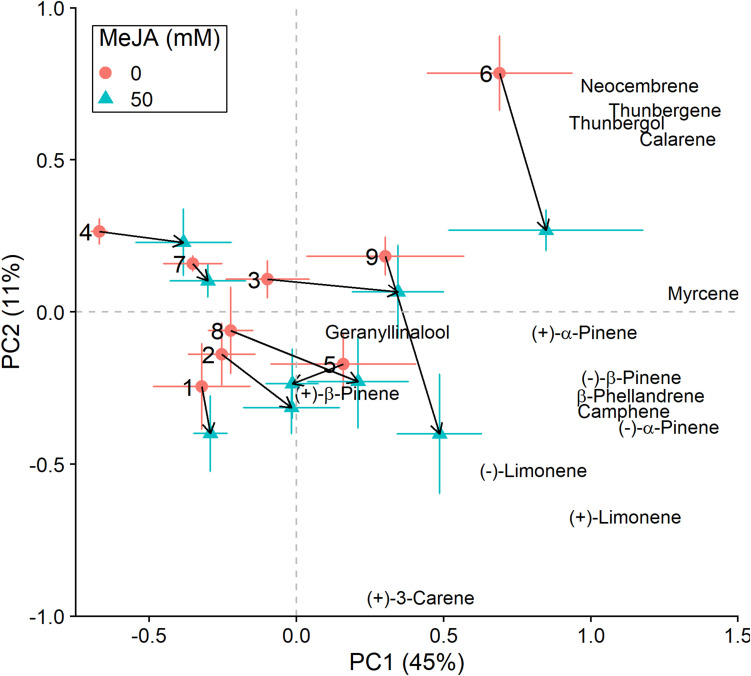
Principal component analysis (PCA) biplots (for non-fungus inoculated plants) of total absolute terpene amounts. Variation along the two main axes of variation (PC 1, PC 2; % variation explained by each axis) is shown for methyl jasmonate-treated (50 mM MeJA, blue filled triangles) and non-treated (0 mM MeJA, orange filled circles) clones of Norway spruce (*P. abies*). Mean PCA score values ± standard error for each clone (1–9) are shown, as well as the direction and distance of change (black arrows) in terpene chemistry between the two MeJA treatments. The position of terpenes indicates PCA loadings (vectors), which illustrate each compound’s correlation with the two axes of variation (see Table 3 for loading values).

In addition to changes in resistance to pine weevil damage and blue-stain fungus infection, we also found differences in terpene chemistry among non-treated and MeJA-treated plants. A total of 15 terpene compounds were identified, including monoterpenes [(+)-α-pinene, (−)-α-pinene, (−)-β-pinene, (+)-β-pinene, (+)-3-carene, (−)-β-phellandrene, (−)-limonene, (+)-limonene, camphene, and myrcene], sesquiterpenes (calarene) and a few diterpenes (neocembrene, thunbergene, thunbergol, and geranyllinalool). Total terpene chemistry (absolute amounts) was significantly different between plants treated with 0 and 50 mM MeJA (Table 2). Treatment with MeJA shifted terpene chemistry mostly across the first axis of variation (PC 1), with larger increases in the amounts of compounds such as (−)-α-pinene, (−)-β-pinene, (+)-limonene, β-phellandrene, camphene, myrcene, and calarene (Figure 3 and Table 3). An increase in (+)-3-carene was observed across the second axis of variation for some clones (e.g., clones 6 and 9, Figure 3). MeJA resulted mostly in quantitative rather than qualitative changes in terpene chemistry, as it had a non-significant effect on the relative proportions of terpene compounds (Supplementary Figure 3 and Supplementary Tables 5, 6). Even though changes in chemistry following MeJA treatment were similar among clones (non-significant MeJA × clone interaction, Table 2 and Supplementary Table 5), total terpene levels differed significantly among clones irrespective of treatment (Figure 3, Supplementary Figure 3, Table 2, and Supplementary Table 5). Among-clone differences explained most of the variation in terpene chemistry rather than MeJA treatment (Table 2 and Supplementary Table 5).

**TABLE 2:** Multivariate analysis of variance (MANOVA) results (DF, degrees of freedom; SS, Sum of Squares; MS, Mean Squares; *F*, *F*-value; *R*^2^, adjusted *R*-squared; and *P*, *p*-value).

	Source	DF	SS	MSS	*F*	*R*^2^	*P*
No fungus exposure	MJ	1	31.83	31.83	3.37	0.02	**0.01**
	Clone	8	584.64	68.58	7.25	0.42	**0.001**
	MJ × clone	8	62.44	7.80	0.83	0.05	0.7
	Residuals	70	662.08	9.46		0.51	
	Total	87	1305.00			1.00	
Exposed to fungus	MJ	1	33.92	33.92	3.50	0.03	**0.01**
	Clone	8	501.60	62.70	6.48	0.39	**0.001**
	MJ × clone	8	86.44	10.81	1.12	0.07	0.28
	Residuals	69	668.06	9.68		0.52	
	Total	86	1290.00			1.00	

*Models examined the effect of Norway spruce (*P. abies*) clone, MeJA treatment (MJ: 0, 50 mM) and their interaction, on absolute terpene amounts when plants had not been (No fungus exposure) or had been exposed to *E. polonica* (Exposed to fungus) following MeJA treatment. Significant effects are in bold (*P* < 0.05).*

**TABLE 3:** Loadings for each compound in Principal components 1 and 2 (PC 1, PC 2) from the PCAs that examined variation in total absolute terpene chemistry (see Figures 3, 4) among Norway spruce (*P. abies*) clones treated with 0 and 50 mM MeJA.

Compound	No fungus exposure		Exposure to fungus	
	PC1	PC2	PC1	PC2
(−)-α-Pinene	0.31	−0.20	0.33	−0.15
(+)-α-Pinene	0.24	−0.04	0.22	−0.13
Camphene	0.28	−0.16	0.22	−0.14
(+)-β-Pinene	0.04	−0.14	0.06	0.33
(−)-β-Pinene	0.28	−0.14	0.31	−0.05
Myrcene	0.34	0.03	0.34	−0.09
(+)-3-Carene	0.10	−0.48	0.22	−0.27
(−)-Limonene	0.20	−0.27	0.26	−0.18
(+)-Limonene	0.28	−0.35	0.31	−0.27
β-Phellandrene	0.29	−0.15	0.31	−0.05
Thunbergene	0.31	0.32	0.26	0.47
Calarene	0.32	0.32	0.27	0.34
Neocembrene	0.29	0.38	0.19	0.36
Geranyllinalool	0.08	−0.04	0.06	0.40
Thunbergol	0.27	0.32	0.29	0.10

*Separate analyses were conducted for plants not exposed (No fungus exposure) and those exposed to *E. polonica* (Exposure to fungus).*

**FIGURE 4:**
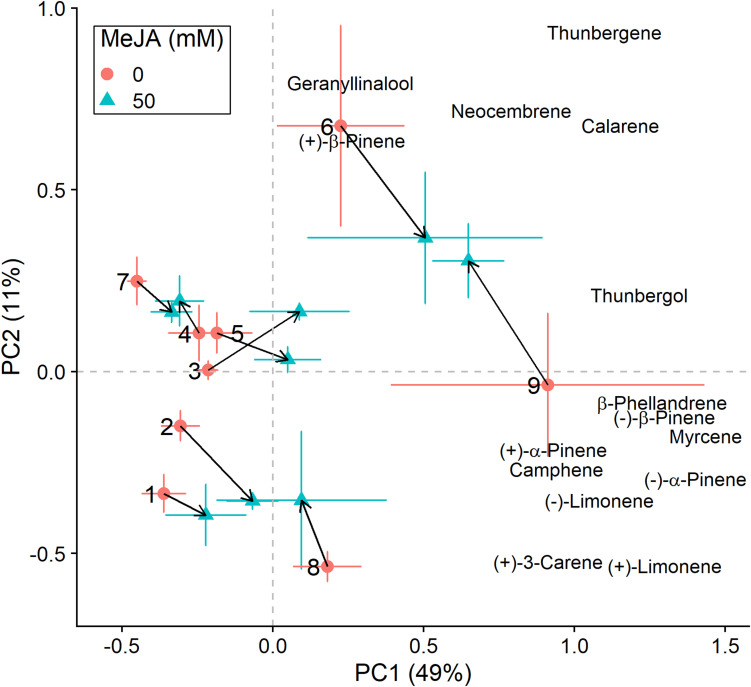
Principal component analysis (PCA) biplots (for *E. polonica*-inoculated plants) of total absolute terpene amounts. Variation along the two main axes of variation (PC 1, PC 2; % variation explained by each axis) is shown for methyl jasmonate-treated (50 mM MeJA, blue filled triangles) and non-treated (0 mM MeJA, orange filled circles) clones of Norway spruce (*P. abies*). Mean PCA score values ± standard error for each clone (1–9) are shown, as well as the direction and distance of change (black arrows) in terpene chemistry between the two MeJA treatments. The position of terpenes indicates PCA loadings (vectors), which illustrate each compound’s correlation with the two axes of variation (see Table 3 for loading values).

We conducted the same analyses as described above also for plants that had received inoculation with *E. polonica*. Treatment with MeJA had a signficant effect on the total concentration of terpenes (Table 2), and it influenced the clones’ positions along the axes of variation differently than when plants were not inoculated with the fungus (Figures 3, 4). Both increases and decreases along PC1 and PC2 were observed depending on the clone. Similar to those not inoculated with the fungus, compounds like (−)-α-pinene, (−)-β-pinene and myrcene increased following MeJA, but changes in compounds such as geranyllinalool and (+)-3-carene were more important for plants responses to the fungus (Figure 4 and Table 3). Again, differences among clones explained most of the variation in terpene chemistry (Table 2). Unlike the effects observed when MeJA occurred alone (without fungal inoculation, Supplementary Figure 3), fungal infection caused qualitative changes in terpene chemistry as indicated by changes in the relative amounts of terpenes (Supplementary Figure 4 and Supplementary Table 5). These relative changes varied per treatment and clone (MeJA × clone interaction, Supplementary Table 5).

## Discussion

Our study showed that treatment with the plant hormone methyl jasmonate (MeJA) increased the resistance of Norway spruce (*P. abies*) clones that were separately exposed to pine weevil (*H. abietis*) damage and blue-stain fungus (*E. polonica*) infection. Treatment with MeJA also changed the terpene chemistry of plants relative to those that were untreated, however, most of the variation in chemistry was explained by differences between Norway spruce clones. Most interestingly, we found that resistance against the pine weevil and blue-stain fungus exhibited a trade-off when comparing the estimates of area debarked and lesion lengths for each clone. Clones that received little pine weevil damage exhibited larger fungal lesion lengths, and vice versa, both constitutively and when treated with MeJA. To our knowledge, this is the first report of such a trade-off.

Treatment with MeJA enhanced Norway spruce resistance to insect and fungal damage when clones were separately exposed to these two pests. On average, plants treated with 50 mM MeJA received 76% less *H. abietis* damage and experienced 55% shorter *E. polonica* lesion lengths relative to untreated control plants. Clones receiving 25 mM MeJA also experienced similar reductions (78% less pine weevil damage and 56% shorter lesion lengths compared to controls). Our results are in line with previous studies on MeJA-induced resistance in *P. abies* and other conifers species. Decreases in pine weevil damage ranging from 50 to 70%, for example, have been shown to occur after consecutive treatment with 10 mM MeJA in Norway spruce seedlings (Chen Y. et al., 2020). For other species such as *Pinus pinaster*, pine weevil damage was reduced by 80% after treatment with 100 mM MeJA (Moreira et al., 2009), and reduced by 30–60% for *P. pinaster*, *P. radiata*, *P. sylvestris*, and *P. abies* seedlings treated with 25 mM MeJA (Zas et al., 2014). MeJA can change the pattern of pine weevil feeding, with weevils inflicting fewer and smaller scars on induced seedlings (Fedderwitz et al., 2016), and resulting in less damage overall. Similarly, treatment with 10 mM MeJA has also been shown to reduce feeding by the bark-feeding beetle *Monochamus alternatus* in *Pinus massoniana* roughly by half compared to non-treated plants (Chen R. et al., 2020). For *E. polonica*, Norway spruce seedlings treated with 100 mM MeJA have been reported to experience 51% shorter lesion lengths than control plants (Krokene et al., 2008). Even mature trees treated with 50 mM MeJA have shown decreased blue-staining of the sapwood (caused by *E. polonica* infection) compared to non-treated trees (15% vs. 70% staining, respectively; Zeneli et al., 2006). Plant resistance to other pathogens, such as *Sphaeropsis sapinea* and *Pythium ultimum* in *P. radiata* and *P. abies*, respectively, has also been shown to be enhanced by exogenous application of MeJA (Kozlowski et al., 1999; Gould et al., 2008).

Even though overall plant resistance was improved, we found that MeJA-mediated changes in resistance occurred to a greater or lesser extent depending on the Norway spruce clone examined. Some clones exhibited high levels of resistance (i.e., received less insect damage or fungal infection) constitutively (0 mM MeJA), and for these clones, MeJA treatment did not result in a large change in area debarked or lesion length (flatter reaction norms from 0 to 50 mM MeJA in Figure 2). Likewise, clones that were less resistant (i.e., received greater insect damage or fungal infection) constitutively, experienced large reductions in pine weevil feeding and shorter fungal lesions (i.e., became more resistant) when treated with MeJA (steeper reaction norms in Figure 2). Thus, not all clones responded equally to MeJA treatment and their degree of MeJA-mediated inducibility differed. In line with these findings, other studies have also found that conifer clones, genotypes, families, provenances, and even different species can vary in their responses to MeJA (Zeneli et al., 2006; Heijari et al., 2008; Semiz et al., 2012; Moreira et al., 2013, 2014; López-Goldar et al., 2018). However, a recent meta-analysis (Howe et al., 2020) found that inverse relationships between constitutive and inducible levels of defense (i.e., individuals with high constitutive defense do not show large changes when induced) against bark beetle damage are often detected at the genotype/family level in common gardens, yet these relationships do not hold at the forest population level. Thus, the scale at which genetic correlations are examined is relevant for their detection and implications. If MeJA is to be implemented as a tool in plant protection, it is important that this variability among individuals or genotypes is considered and quantified. For example, screening for variation in the degree of inducibility in material from tree breeding populations or from various provenances, should be conducted.

In addition to the effects of MeJA treatment on overall resistance, its effect on terpene chemistry was also investigated. Plants treated with MeJA exhibited an increase in the amounts of terpenes such as α-pinene, β-pinene, limonene, β-phellandrene, camphene, calarene, myrcene and even (+)-3-carene. Terpene accumulation following MeJA treatment has been previously described to occur in Norway spruce (Franceschi et al., 2002; Martin et al., 2002; Erbilgin et al., 2006; Zulak et al., 2009; Zhao et al., 2010), and also in other conifer species (e.g., Hudgins et al., 2003; Erbilgin and Colgan, 2012; Pham et al., 2014; Lundborg et al., 2019; Chen R. et al., 2020). In line with our findings, Zhao et al. (2010) and Lundborg et al. (2016b) found that α-pinene and limonene respond strongly to treatment with MeJA in Norway spruce. Similarly, myrcene and β-pinene have been shown to increase after treatment with MeJA in mature Jack pine trees (*Pinus banksiana*) and Chinese white pine (*Pinus armandi*) saplings (Erbilgin and Colgan, 2012; Pham et al., 2014). We also found that MeJA treatment resulted in changes in absolute terpene amounts, but did not have a significant effect on their relative proportions (Supplementary Table 5). Thus, MeJA resulted in quantitative rather than qualitative changes in terpene chemistry, which is also in line with previous findings (Zhao et al., 2010).

For plants that were inoculated with *E. polonica* instead of an uninfected agar plug, we observed similar effects of MeJA treatment, yet more variation in responses among clones (Figure 4). Also, qualitative changes in terpene chemistry occurred (Supplementary Table 5). Compounds such as (+)-3-carene and geranyllinalool varied more strongly among clones (across the first axis of variation) when MeJA treatment and fungal infection co-occurred. This is consistent with previous studies which have found that (+)-3-carene is often induced following fungal infection (Croteau et al., 1987; Fäldt et al., 2006; Zhao et al., 2010), and that both compounds appear to play a role in differential susceptibility of Norway spruce to *E. polonica* and *Heterobasidion parviporum* infection (Danielsson et al., 2011; Axelsson et al., 2020). Moreover, (+)-3-carene has also been associated with conifer resistance against insects. For example, it is important for Sitka spruce (*Picea sitchensis*) resistance against the white pine weevil (*Pissodes strobi*; Robert et al., 2010), in *P. sylvestris* against the pine sawfly *Diprion pini* (Pasquier-Barre et al., 2001), and in lodgepole pine (*Pinus contorta*) against the Douglas-fir pitch moth (*Synanthedon novaroensis*; Rocchini et al., 2000). Lundborg et al. (2016b) also found that (+)-3-carene appears to be a feeding deterrent to the pine weevil (*H. abietis*), however, it seems to be induced by MeJA to a lesser extent in *P. abies* compared to *P. sylvestris* (Lundborg et al., 2016a). Overall, the changes we observed in terpene chemistry are consistent with ours and others’ previous work, and corroborate that MeJA induces changes in defensive chemistry that likely influence Norway spruce resistance to insect damage and fungal infection.

Despite the significant effect of MeJA on terpene compounds, treatment explained little variation in plant defensive chemistry relative to clone (2% vs. 42%, respectively, Table 2). Norway spruce clones responded in a similar way to MeJA treatment (non-significant MeJA × clone interaction, Table 2), however, differences among clones were most important for determining terpene composition. This in contrast to the study by López-Goldar et al. (2018) where MeJA treatment explained 31% of the variation in defensive compounds, while family explained 2%, in *P. pinaster*. Yet, it is in line with other studies in which variation in defensive chemistry has been to a large extent explained by among family (or genotype/clone) differences (e.g., Havill and Raffa, 1999; Ott et al., 2011; Axelsson et al., 2020). Our results from terpene chemistry analyses are consistent with our results on insect damage and fungal lesion lengths, for which we also found that differences among clones had a strong effect on the response variables (Table 1). All in all, if MeJA is to be implemented as a plant protection tool, these findings reiterate firstly the importance of quantifying among-clone (or family) variation in defensive responses. And secondly, evaluating the relative importance of constitutive and MeJA-induced responses among clones, and the relationship with actual resistance (i.e., change in damage or infection levels).

Lastly, but most interestingly, we also found that damage by *H. abietis* negatively correlated with fungal infection by *E. polonica* both when treated with 0 and 50 mM MeJA. Clones that were constitutively more resistant to *H. abietis*, exhibited lower resistance against *E. polonica* and vice versa when clones were separately exposed to these two pests. Likewise, those genotypes showing a high degree of MeJA-inducibility for insect damage (greatest change in area debarked from 0 to 50 mM MeJA) showed little change in fungal infection when induced. Thus, there is a trade-off between resistance to *E. polonica* and *H. abietis*. Few studies have examined the relationship between resistance to both insect and fungal pests in conifers, and using the same clones. To our knowledge, this is the first time a trade-off between pine weevil damage and a fungal infection has been reported. Relationships among different fungal pathogens are more often studied, and in these studies resistance or susceptibility to fungal infection have been shown to correlate positively or negatively with each other (e.g., Bonello et al., 2008; Xu et al., 2018; Hurel et al., 2019). For instance, infection by *Heterobasidion annosum* in the basal stem of *Pinus pinea* reduced the concentration of total terpenoids in the shoots in response to *Diplodia pinea* inoculation relative to those found in healthy shoots (Bonello et al., 2008). Lower concentrations of terpenes were found in shoots more susceptible to *D. pinea* (i.e., those with greater lesion sizes), indicating that higher resistance to *H. annosum* came at the cost of resistance to *D. pinea* (Bonello et al., 2008). In our study, MeJA treatment increased resistance to both insect and fungal damage, thus it is less likely that a negative correlation among resistances is due to different defense pathways being involved. Also, as described above, many of the terpene compounds that reduce fungal infection have also been shown to play a role in deterring insect feeding (e.g., Zhao et al., 2010; Lundborg et al., 2016b). Note, however, that we evaluated resistance to each pest separately in clonal individuals (i.e., infection and pine weevil damage did not co-occur). It is possible that the clones examined differed in other unmeasured traits (morphological, physiological or chemical), which confer high constitutive resistance to insect or fungal damage. We are currently unable to discern underlying mechanisms behind this trade-off, but it certainly deserves further attention.

Even though pine weevil damage and infection by *E. polonica* do not co-occur naturally, a negative correlation among resistance to each of these pests could potentially constrain the availability of suitable Norway spruce genotypes for tree breeding programs. Current breeding programs in Sweden and other Nordic countries are interested in identifying clones or families with resistance to pine weevil damage, but also resistance to attack by the spruce bark beetle and infection by *E. polonica* (Steffenrem et al., 2016; Zas et al., 2017; Puentes et al., 2018; Axelsson et al., 2020). Yet, the negative correlation documented in our study does not necessarily pose a hinder for selection of such families. Clones that are constitutively more resistant to *E. polonica* should show high MeJA-induced resistance to *H. abie*tis damage and vice versa. In other words, Norway spruce clones that are constitutively more resistant to one of the pests, should show greater induced resistance against the other. One alternative could be the selection of clones/families that exhibit high MeJA-mediated resistance against pine weevil damage and are constitutively more resistant to fungal infection. Protection against pine weevil damage is necessary for only a few years after planting, since pine weevils cause high mortality only at the seedling stage. In terms of applicability, several studies have shown that treating seedlings with MeJA can be compatible with nursery practices (Fedderwitz et al., 2019; Chen Y. et al., 2020). Infection by *E. polonica* occurs when mature trees are attacked by the spruce bark beetle, and it would be important for these trees to be constitutively more resistant to infection. Note that we have identified this negative correlation among resistances at an early plant stage, in separate clonal individuals and with a limited number of clones. This relationship could, thus, be different for mature Norway spruce trees that were damaged early in life by pine weevils and later become infected by *E. polonica*. Nonetheless, if breeding for increased resistance is of interest, this trade-off should be taken into consideration.

So far, we have discussed our results assuming that MeJA acts as an elicitor that results in defense induction, which has been supported by previous studies (e.g., Hudgins et al., 2003; Krokene et al., 2008). It is important to note that recently, MeJA was also found to be able to act as a priming stimulus and need not always result in full direct induction of defenses. Mature Norway spruce trees treated with MeJA did not show any change in terpene levels 14 days after treatment, yet they experienced increased resistance to spruce bark beetle attack relative to untreated controls, 65 days after treatment (Mageroy et al., 2020a). MeJA treatment can lead to the formation of an immunological-like memory, and a second stimulus (wounding, feeding, and infection) allows plants to recall this memory and super-induce defenses (Mageroy et al.,2020a,b). In our study, we observed both changes in terpene chemistry and enhanced resistance following MeJA treatment. Treatment occurred in April/May and plants were exposed to the insects/fungus in July, while samples for chemical analyses were taken in August. The effects of MeJA on terpenes and resistance can change with time, but can still be observed at 1 month and even 1 year after treatment (Martin et al., 2002; Miller et al., 2005; Zhao et al., 2010; Zas et al., 2014). Thus, our experimental timings are in line with when effects can be detected. However, since our study design was not intended for discerning the underlying mechanisms of MeJA treatment, we are unable to say whether priming or direct induction of defenses occurred.

## Main Conclusion

We conclude that MeJA treatment can change Norway spruce terpene chemistry, and increase resistance to insect damage by *H. abietis* and fungal infection by *E. polonica*. However, for Norway spruce genotypes that are constitutively more resistant to insect damage or fungal infection, treatment with MeJA may not result in larger additional changes in resistance. Differences among clones appear to be most important for changes in defensive chemistry and degree of MeJA-mediated responses. Interestingly, we also found a trade-off between resistance to insect and fungal damage when examining estimates per clone, but this does not necessarily entail negative consequences for overall plant defense or hinder selection of the most resistant trees. Norway spruce clones that are constitutively more resistant to one of the pests, should show greater induced resistance against the other. Thus, treatment with MeJA should result in enhanced resistance to one of the pests, without compromising resistance to the other. Future studies should quantify the relative importance of constitutive vs. induced responses to various types of pests, and examine the generality of negative correlations among resistances in a larger number of clones, as well as in other conifer species.

## Data Availability Statement

The raw data supporting the conclusions of this article will be made available by the authors upon request, without undue reservation.

## Author Contributions

A-KB-K, TZ, NB, and LL conceived and planned the experiments. NB, TZ, and LL conducted the experiments and collected the data. TZ and LL performed chemical analyses. AP conducted the statistical analyses of the data and wrote the manuscript with input from co-authors. All authors contributed to the article and approved the submitted version.

## Conflict of Interest

The authors declare that the research was conducted in the absence of any commercial or financial relationships that could be construed as a potential conflict of interest.
