# Supplementary material for: Variation in Methyl Jasmonate-Induced Defense Among Norway Spruce Clones and Trade-Offs in Resistance Against a Fungal and an Insect Pest
Source: Front Plant Sci. 2021 May 24;12:678959. doi: 10.3389/fpls.2021.678959 (PMC8182065; doi:10.3389/fpls.2021.678959)
Supplement: Supplementary file 1 [file Data_Sheet_1.pdf]

# *Supplementary Material*

## 1 Supplementary Figures and Tables

**Puentes et al.** - Variation in methyl jasmonate-induced defense among Norway spruce clones and trade-offs in resistance against a fungal and an insect pest

Tables: 7

Figures: 4

## 1.1 Supplementary Figures

**Figure S1.** Picture showing some of the Norway spruce (*P. abies*) plants used in the experiment. The second MeJA treatment had been applied on May 14th (one plant for each of the treatments is shown: from the left, 0, 25 and 50 mM MeJA), and the picture was taken the day of planting (May 21st) in the semi-field experiment.

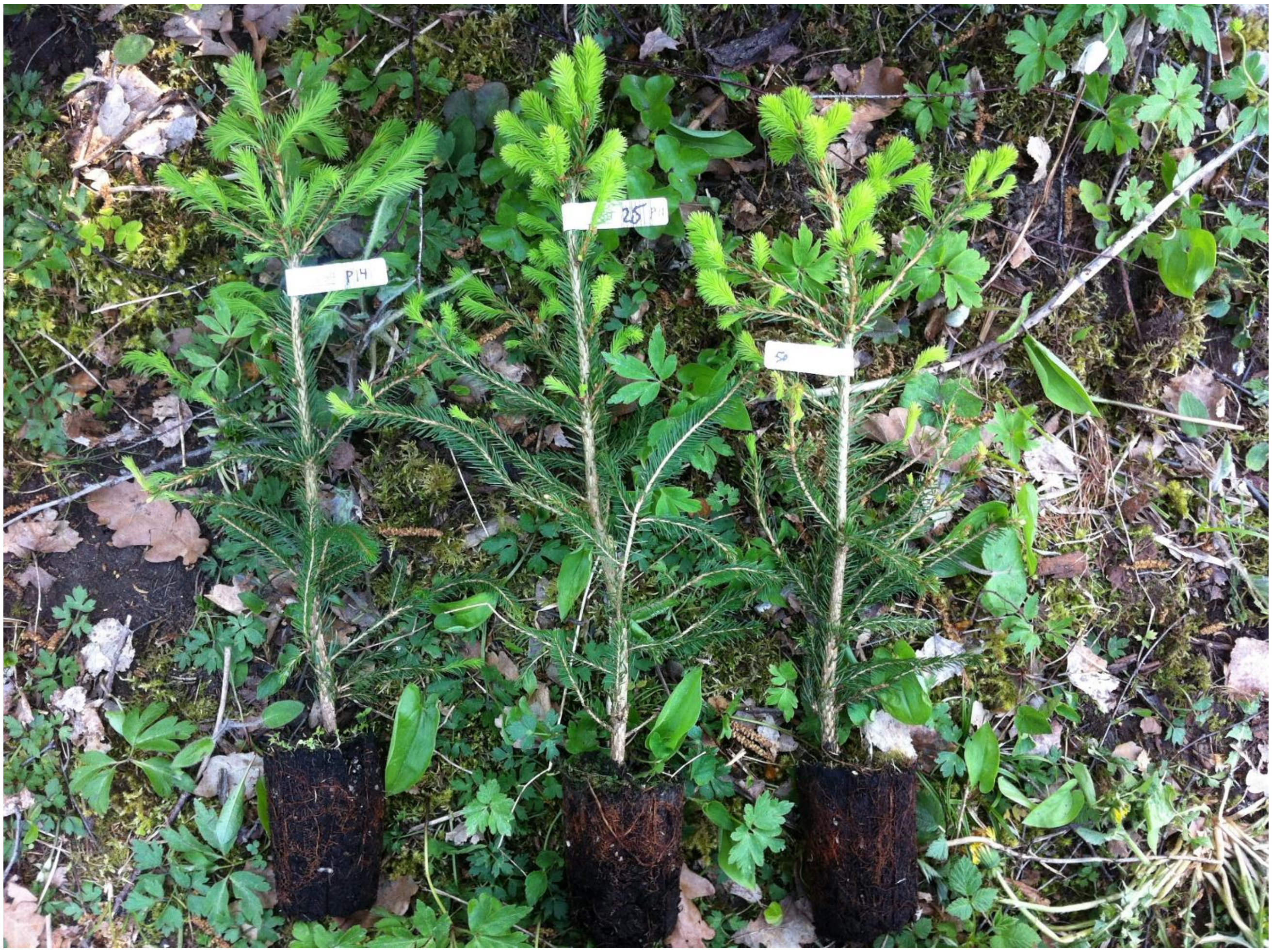

**Figure S2.** Mean ± standard error **(a)** plant height (cm), **(b)** stem diameter (mm), and **(c)** top shoot length (mm) per clone for Norway spruce (*P. abies*) plants treated with 0, 25 or 50 mM methyl Jasmonate (MeJA). Clone number 1 did not have any plants in the 50 mM MeJA treatment (see Materials and Methods for justification and sample sizes per clone and treatment combination).

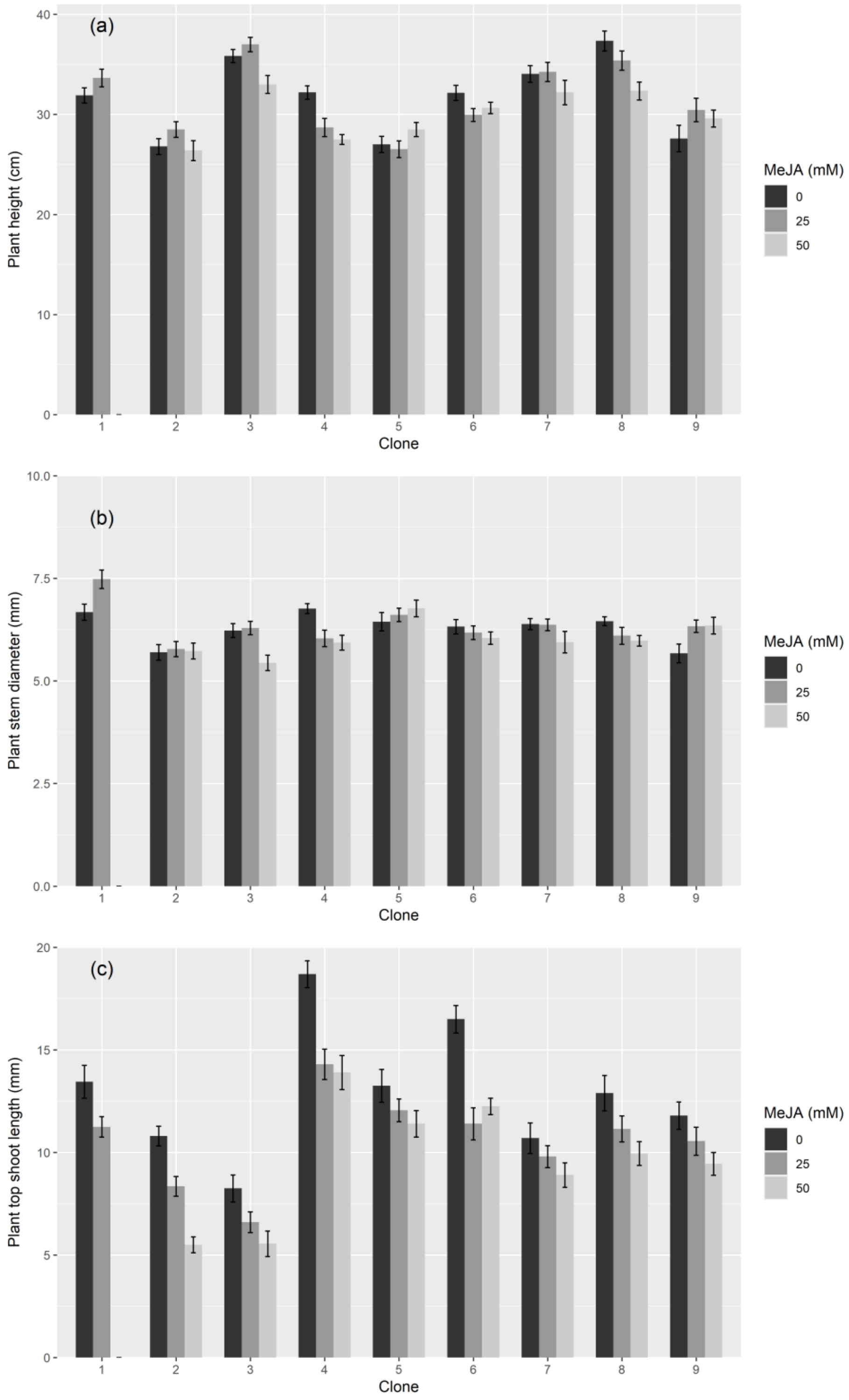

**Figure S3.** Principal component analysis (PCA) biplots (for non-fungus inoculated plants) of relative terpene amounts. Variation along the two main axes of variation (PC 1, PC 2; % variation explained by each axis) is shown for methyl jasmonate-treated (50 mM MeJA, blue filled triangles) and non-treated (0 mM MeJA, orange filled circles) clones of Norway spruce (*P. abies*). Mean PCA score values (± standard errors) for each clone (1-9) are shown, as well as the direction and distance of change (black arrows) in terpene chemistry between the two MeJA treatments. The position of terpenes indicates PCA loadings (vectors), which illustrate each compound's correlation with the two axes of variation (see Table S6 for loading values).

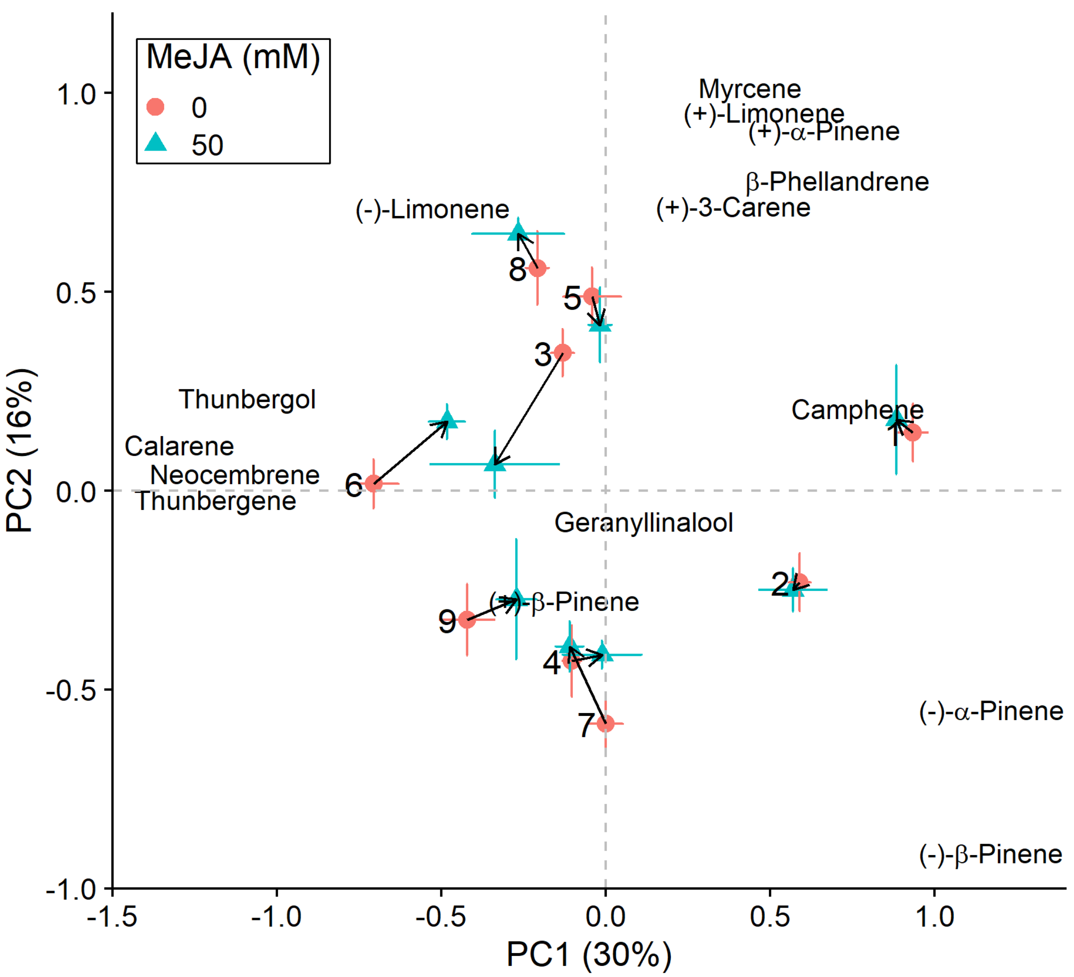

**Figure S4.** Principal component analysis (PCA) biplots (for *E. polonica*-inoculated plants) of relative terpene amounts. Variation along the two main axes of variation (PC 1, PC 2; % variation explained by each axis) is shown for methyl jasmonate-treated (50 mM MeJA, blue filled triangles) and non-treated (0 mM MeJA, orange filled circles) clones of Norway spruce (*P. abies*). Mean PCA score values (± standard errors) for each clone (1-9) are shown, as well as the direction and distance of change (black arrows) in terpene chemistry between the two MeJA treatments. The position of terpenes indicates PCA loadings (vectors), which illustrate each compound's correlation with the two axes of variation (see Table S6 for loading values).

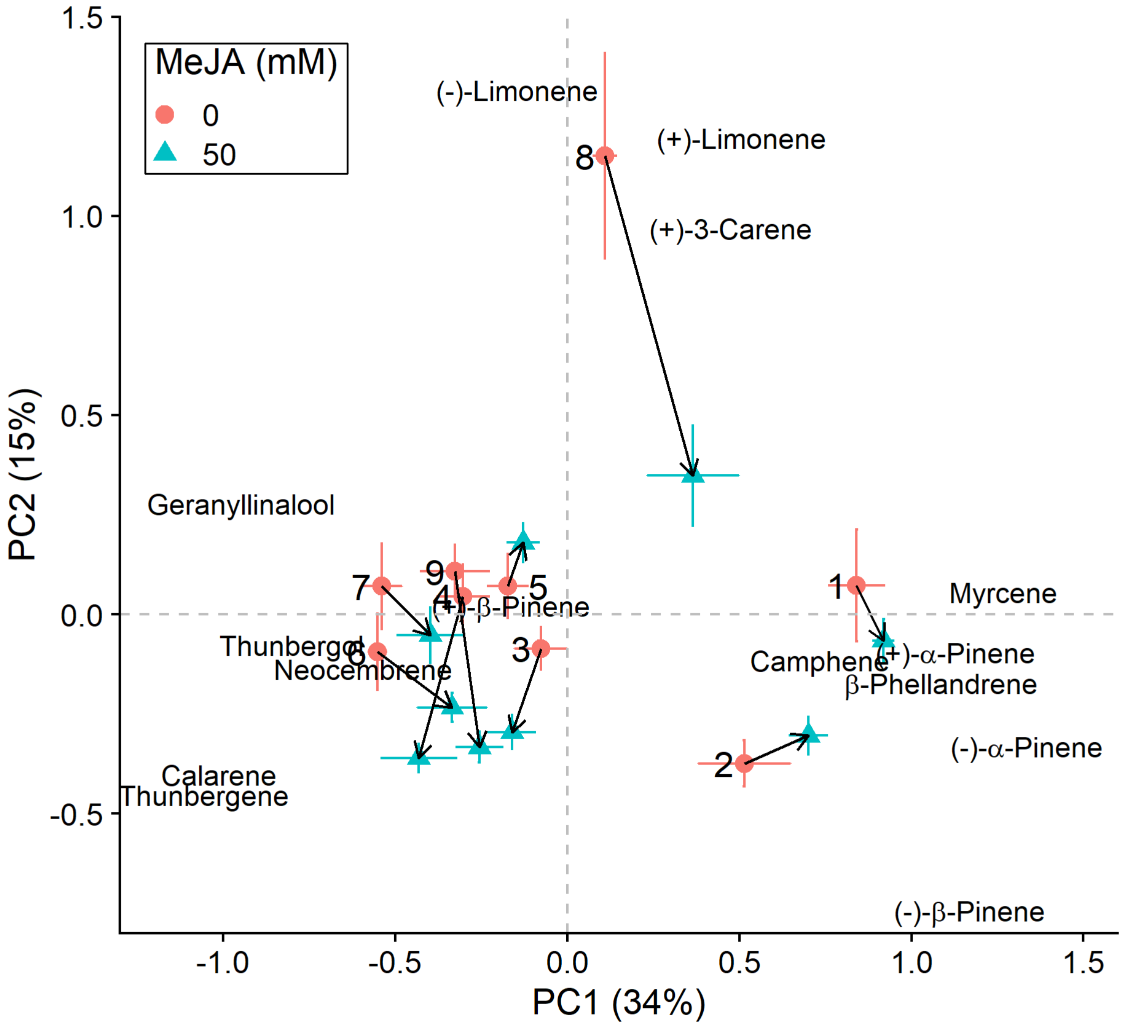

## 1.2 Tables

**Table S1.** Krutzch index indicating the developmental stage of Norway spruce (*P. abies*) clones before MeJA treatment was conducted. The index ranges between 0 to 8 and describes terminal bud development on the uppermost whorl of branches. 0 = Dormant buds, 1 = Buds slightly swollen, needles below buds bent backwards and outwards, 2 = Buds swollen, green to grey-green in color, bud scales still closed, 3 = Burst of bud scales, tips of needles emerging. See Krutzch (1973) for the description of remaining index values (4-8).

| Clone # | Index |
|---|---|
| 1 | 3 |
| 2 | 0 |
| 3 | 0 |
| 4 | 3 |
| 5 | 2 |
| 6 | 3 |
| 7 | 1 |
| 8 | 0 |
| 9 | 0 |

**Table S2.** Results from contrasts between treatments, obtained from models examining the effect of MeJA (MJ: 0, 25 or 50 mM) on area debarked ($mm^2$) by pine weevils (*H. abietis*) or lesion lengths (mm) inflicted by the blue-stain fungus (*E. polonica*) on Norway spruce (*P. abies*) clones. Models included the main effect and interaction of MeJA and clone, experimental enclosure or sub-plot for lesion length, and plant height as a covariate (for area debarked only). Estimate of differences between treatment means (Estimate), standard error of the difference (SE), degrees of freedom (DF), t-ratio, and p-value (P) are presented. Note that for clone 1, no plants in the 50 mM MeJA treatment were included in the experiment (see Materials and Methods).

Table in next page.

Table S2

| Variable | Clone | Contrast | Estimate | SE | DF | t-ratio | P |
|---|---|---|---|---|---|---|---|
| Area debarked (mm$^2$) | 1 | 0 vs MJ25 | 99.0 | 21.2 | 337 | 4.6 | <0.0001 |
| | 2 | 0 vs MJ25 | 75.4 | 19.9 | 448 | 3.8 | 0.0002 |
| | | 0 vs MJ50 | 76.1 | 19.8 | 448 | 3.8 | 0.0001 |
| | | MJ25 vs MJ50 | 0.7 | 6.2 | 448 | 0.1 | 0.9 |
| | 3 | 0 vs MJ25 | 68.7 | 16.4 | 448 | 4.2 | <0.0001 |
| | | 0 vs MJ50 | 73.1 | 16.6 | 448 | 4.4 | <0.0001 |
| | | MJ25 vs MJ50 | 4.4 | 5.3 | 448 | 0.8 | 0.4 |
| | 4 | 0 vs MJ25 | 58.8 | 15.4 | 448 | 3.8 | 0.0002 |
| | | 0 vs MJ50 | 49.2 | 16.2 | 448 | 3.0 | 0.003 |
| | | MJ25 vs MJ50 | -9.6 | 7.9 | 448 | -1.2 | 0.2 |
| | 5 | 0 vs MJ25 | 41.5 | 15.7 | 448 | 2.6 | 0.009 |
| | | 0 vs MJ50 | 35.3 | 16.2 | 448 | 2.2 | 0.03 |
| | | MJ25 vs MJ50 | -6.1 | 10.5 | 448 | -0.6 | 0.6 |
| | 6 | 0 vs MJ25 | 56.5 | 13.6 | 448 | 4.2 | <0.0001 |
| | | 0 vs MJ50 | 55.3 | 13.6 | 448 | 4.1 | 0.0001 |
| | | MJ25 vs MJ50 | -1.2 | 4.00 | 448 | -0.3 | 0.76 |
| | 7 | 0 vs MJ25 | 36.9 | 12.4 | 448 | 3.0 | 0.003 |
| | | 0 vs MJ50 | 38.4 | 12.4 | 448 | 3.1 | 0.002 |
| | | MJ25 vs MJ50 | 1.6 | 6.9 | 448 | 0.2 | 0.8 |
| | 8 | 0 vs MJ25 | 15.2 | 8.4 | 448 | 1.82 | 0.07 |
| | | 0 vs MJ50 | 14.4 | 8.7 | 448 | 1.7 | 0.09 |
| | | MJ25 vs MJ50 | -0.8 | 6.1 | 448 | -0.1 | 0.90 |
| | 9 | 0 vs MJ25 | 10.7 | 7.8 | 448 | 1.4 | 0.2 |
| | | 0 vs MJ50 | 8.3 | 8.0 | 448 | 1.0 | 0.3 |
| | | MJ25 vs MJ50 | -2.4 | 5.6 | 448 | -0.4 | 0.7 |
| Lesion length (mm) | 1 | 0 vs MJ25 | 2.8 | 1.5 | 66 | 1.9 | 0.07 |
| | 2 | 0 vs MJ25 | 1.8 | 0.9 | 88 | 1.9 | 0.05 |
| | | 0 vs MJ50 | 1.2 | 0.9 | 88 | 1.2 | 0.2 |
| | | MJ25 vs MJ50 | -0.7 | 0.8 | 88 | -0.9 | 0.4 |
| | 3 | 0 vs MJ25 | 2.5 | 1.7 | 88 | 1.4 | 0.16 |
| | | 0 vs MJ50 | 3.6 | 1.7 | 88 | 2.1 | 0.03 |
| | | MJ25 vs MJ50 | 1.1 | 1.4 | 88 | 0.8 | 0.42 |
| | 4 | 0 vs MJ25 | 5.5 | 1.8 | 88 | 3.1 | 0.003 |
| | | 0 vs MJ50 | 4.6 | 1.8 | 88 | 2.5 | 0.01 |
| | | MJ25 vs MJ50 | -0.9 | 1.0 | 88 | -0.9 | 0.4 |
| | 5 | 0 vs MJ25 | 6.4 | 1.8 | 88 | 3.5 | 0.0008 |
| | | 0 vs MJ50 | 6.0 | 1.8 | 88 | 3.3 | 0.001 |
| | | MJ25 vs MJ50 | -0.4 | 0.9 | 88 | -0.4 | 0.7 |
| | 6 | 0 vs MJ25 | 1.4 | 1.4 | 88 | 1.0 | 0.3 |
| | | 0 vs MJ50 | 3.2 | 1.3 | 88 | 2.5 | 0.01 |
| | | MJ25 vs MJ50 | 1.8 | 1.1 | 88 | 1.7 | 0.09 |
| | 7 | 0 vs MJ25 | 1.5 | 0.9 | 88 | 1.6 | 0.1 |
| | | 0 vs MJ50 | 0.7 | 1.0 | 88 | 0.7 | 0.5 |
| | | MJ25 vs MJ50 | -0.8 | 0.8 | 88 | -0.9 | 0.4 |
| | 8 | 0 vs MJ25 | 10.6 | 2.4 | 88 | 4.4 | <0.0001 |
| | | 0 vs MJ50 | 10.6 | 2.4 | 88 | 4.4 | <0.0001 |
| | | MJ25 vs MJ50 | -0.02 | 0.8 | 88 | -0.02 | 0.9 |
| | 9 | 0 vs MJ25 | 13.3 | 2.8 | 88 | 4.7 | <0.0001 |
| | | 0 vs MJ50 | 12.7 | 2.9 | 88 | 4.4 | <0.0001 |
| | | MJ25 vs MJ50 | -0.6 | 0.9 | 88 | -0.7 | 0.5 |

**Table S3.** Estimated means (from models presented in Table 1 in the main text) ± standard error for area debarked ($mm^2$) by pine weevils (*H. abietis*) and lesion lengths (mm) inflicted by the blue-stain fungus (*E. polonica*) on Norway spruce (*P. abies*) clones (1-9) that were treated with 0 mM and 50 mM methyl jasmonate (MeJA). These estimated means are depicted in Figure 1 in the main text.

| Clone | 0 mM MeJA | | 50 mM MeJA | |
|---|---|---|---|---|
| | Area debarked ($mm^2$) | Lesion length (mm) | Area debarked ($mm^2$) | Lesion length (mm) |
| 1 | 42.47 ± 1.26 | 5.61 ± 1.24 | 8.07 ±1.39 | 2.84 ± 1.23 |
| 2 | 32.33 ± 1.31 | 4.69 ± 1.23 | 4.15 ± 1.53 | 3.60 ± 1.23 |
| 3 | 27.53 ± 1.29 | 8.48 ± 1.25 | 2.82 ± 1.56 | 5.04 ± 1.28 |
| 4 | 25.75 ± 1.30 | 8.70 ± 1.26 | 8.37 ± 1.41 | 4.44 ± 1.26 |
| 5 | 22.61 ± 1.34 | 9.71 ± 1.23 | 9.73 ± 1.40 | 4.13 ± 1.23 |
| 6 | 20.76 ± 1.32 | 6.32 ± 1.23 | 2.34 ± 1.62 | 3.41 ± 1.28 |
| 7 | 17.64 ± 1.33 | 4.20 ± 1.26 | 4.88 ± 1.47 | 3.61 ± 1.28 |
| 8 | 9.18 ± 1.38 | 13.39 ± 1.23 | 4.70 ± 1.46 | 3.57 ± 1.24 |
| 9 | 7.64 ± 1.45 | 16.01 ± 1.23 | 4.34 ± 1.49 | 4.27 ± 1.23 |

**Table S4.** Analysis of variance (SS: Sum of Squares, DF: degrees of freedom, F: F-value, P: p-value) results from models examining the effects of MeJA (MJ: 0, 25 or 50 mM) on plant height (cm), top shoot length (mm) and plant diameter (mm) of Norway spruce (*P. abies*) clones. Models included the main effect and interaction of MeJA and clone, and experimental enclosure (Block) as explanatory factors. Clone 1 was not included in these models since plants in the 50 mM MeJA treatment were not included in the experiment (see Materials and Methods). Significant effects are in bold ($P < 0.05$).

| Variable | Source | SS | DF | F | P |
|---|---|---|---|---|---|
| Plant height (cm) | MJ | 233.1 | 2 | 7.7 | **0.0005** |
| | Clone | 4365.1 | 7 | 41.3 | **<0.0001** |
| | MJ x clone | 695.3 | 14 | 3.3 | **<0.0001** |
| | Block | 93.5 | 3 | 2.1 | 0.1 |
| | Residuals | 6800.0 | 450 | | |
| Top shoot length (mm) | MJ | 902.0 | 2 | 56.9 | **<0.0001** |
| | Clone | 3334.2 | 7 | 60.1 | **<0.0001** |
| | MJ x clone | 248.5 | 14 | 2.2 | **0.006** |
| | Block | 136.8 | 3 | 5.8 | 0.0007 |
| | Residuals | 3568.3 | 450 | | |
| Diameter (mm) | MJ | 4.4 | 2 | 3.5 | **0.03** |
| | Clone | 25.3 | 7 | 5.7 | **<0.0001** |
| | MJ x clone | 28.8 | 14 | 2.8 | **0.0005** |
| | Block | 9.2 | 3 | 4.9 | 0.002 |
| | Residuals | 283.8 | 450 | | |

**Table S5.** Multivariate analysis of variance (MANOVA) results (DF: degrees of freedom, SS: Sum of Squares, MS: Mean Squares, F: F-value, $R^2$: adjusted R-squared, P: p-value). Models examined the effect of Norway spruce (*P. abies*) clone, MeJA treatment (MJ: 0, 50 mM) and their interaction, on relative terpene amounts when plants had not been (No fungus exposure) and had been exposed to *E. polonica* (Exposed to fungus) following MeJA treatment. Significant effects are in bold (P < 0.05).

| | **Source** | **DF** | **SS** | **MSS** | **F** | **$R^2$** | **P** |
|---|---|---|---|---|---|---|---|
| No fungus exposure | MJ | 1 | 12.49 | 12.49 | 1.59 | 0.01 | 0.1 |
| | Clone | 8 | 682.07 | 85.26 | 10.85 | 0.52 | **0.001** |
| | Clone x MJ | 8 | 60.57 | 7.58 | 0.96 | 0.05 | 0.5 |
| | Residuals | 70 | 549.87 | 7.86 | | 0.42 | |
| | Total | 87 | 1305.00 | | | 1.00 | |
| Exposed to fungus | MJ | 1 | 39.79 | 39.80 | 6.16 | 0.03 | **0.001** |
| | Clone | 8 | 715.53 | 89.44 | 13.85 | 0.55 | **0.001** |
| | MJ x clone | 8 | 89.07 | 11.13 | 1.72 | 0.07 | **0.004** |
| | Residuals | 69 | 445.60 | 6.46 | | 0.34 | |
| | Total | 86 | 1290.00 | | | 1.00 | |

**Table S6.** Loadings for each compound in Principal components 1 and 2 (PC 1, PC 2) from the PCAs that examined variation in relative terpene chemistry (see Figures S3 and S4) among Norway spruce (*P. abies*) clones treated with 0 and 50 mM MeJA. Separate analyses were conducted for plants not exposed (No fungus exposure) and those exposed to *E. polonica* (Exposure to fungus).

| Compound | No fungus exposure | | Exposure to fungus | |
|---|---|---|---|---|
| | PC1 | PC2 | PC1 | PC2 |
| (-)-α-Pinene | 0.36 | -0.23 | 0.38 | -0.15 |
| (+)-α-Pinene | 0.20 | 0.39 | 0.32 | -0.04 |
| Camphene | 0.23 | 0.08 | 0.21 | -0.05 |
| (+)-β-Pinene | -0.04 | -0.12 | -0.05 | 0.006 |
| (-)-β-Pinene | 0.36 | -0.38 | 0.33 | -0.33 |
| Myrcene | 0.13 | 0.42 | 0.36 | 0.02 |
| (+)-3-Carene | 0.12 | 0.31 | 0.14 | 0.42 |
| (-)-Limonene | -0.16 | 0.30 | -0.04 | 0.57 |
| (+)-Limonene | 0.15 | 0.40 | 0.14 | 0.52 |
| β-Phellandrene | 0.22 | 0.32 | 0.31 | -0.05 |
| Thunbergene | -0.36 | -0.005 | -0.30 | -0.19 |
| Calarene | -0.40 | 0.05 | -0.29 | -0.18 |
| Neocembrene | -0.34 | 0.02 | -0.17 | -0.06 |
| Geranyllinalool | 0.03 | -0.03 | -0.27 | 0.12 |
| Thunbergol | -0.33 | 0.09 | -0.23 | -0.03 |

**Table S7.** Results from Pearson product-moment correlations ($r$ = Pearson correlation coefficient; P = p-value) between estimated means per clone (n = 9) of area debarked ($mm^2$) (or lesion length, mm) and absolute amounts of each terpene compound (or the sum of all terpenes) (μg $g^{-1}$ dry wt). Estimates of area debarked and lesion lengths for each clone were obtained from models presented in Table 1 in the main text. These estimates were correlated to the average absolute terpene amounts of each compound (or the sum of all terpenes) per clone, both under the constitutive and induced state and (0 and 50 mM MeJA respectively) and without fungal inoculation. Significant correlations are in bold (P < 0.05).

| Compound | 0 mM MeJA | | | | 50 mM MeJA | | | |
|---|---|---|---|---|---|---|---|---|
| | Area debarked ($mm^2$) | | Lesion length (mm) | | Area debarked ($mm^2$) | | Lesion length (mm) | |
| | *r* | P | *r* | P | *r* | P | *r* | P |
| (-)-α-Pinene | -0.44 | 0.24 | **0.75** | **0.02** | -0.55 | 0.12 | -0.16 | 0.69 |
| (+)-α-Pinene | 0.04 | 0.93 | 0.22 | 0.56 | -0.36 | 0.34 | -0.52 | 0.15 |
| Camphene | 0.05 | 0.90 | 0.64 | 0.06 | **-0.82** | **0.007** | -0.14 | 0.72 |
| (+)-β-Pinene | -0.31 | 0.41 | -0.18 | 0.65 | -0.12 | 0.76 | -0.21 | 0.60 |
| (-)-β-Pinene | -0.09 | 0.82 | 0.55 | 0.13 | -0.55 | 0.13 | -0.17 | 0.67 |
| Myrcene | 0.05 | 0.90 | **0.79** | **0.01** | **-0.74** | **0.02** | -0.3 | 0.43 |
| (+)-3-Carene | -0.2 | 0.61 | 0.57 | 0.11 | 0.09 | 0.82 | -0.64 | 0.06 |
| (-)-Limonene | **-0.81** | **0.009** | **0.93** | **0.00** | -0.21 | 0.59 | -0.05 | 0.89 |
| (+)-Limonene | -0.31 | 0.42 | **0.91** | **0.001** | -0.28 | 0.47 | -0.43 | 0.25 |
| β-Phellandrene | 0.18 | 0.65 | **0.72** | **0.03** | **-0.74** | **0.02** | -0.29 | 0.44 |
| Thunbergene | -0.46 | 0.21 | 0.45 | 0.22 | **-0.78** | **0.01** | 0.24 | 0.54 |
| Calarene | -0.48 | 0.19 | 0.52 | 0.15 | **-0.80** | **0.009** | 0.31 | 0.41 |
| Neocembrene | -0.41 | 0.27 | 0.54 | 0.13 | -0.55 | 0.13 | -0.13 | 0.75 |
| Geranyllinalool | 0.09 | 0.83 | -0.16 | 0.68 | -0.35 | 0.35 | 0.09 | 0.81 |
| Thunbergol | -0.36 | 0.34 | **0.79** | **0.01** | **-0.68** | **0.04** | 0.23 | 0.55 |
| Sum of terpenes | -0.36 | 0.35 | **0.71** | **0.03** | **-0.80** | **0.01** | -0.02 | 0.97 |
